# Supplementary material for: End Sequence Analysis Toolkit (ESAT) expands the extractable information from single-cell RNA-seq data
Source: Genome Res. 2016 Oct;26(10):1397–410. doi: 10.1101/gr.207902.116 (PMC5052061; doi:10.1101/gr.207902.116)
Supplement: Supplemental Material [file supp_gr.207902.116_Supp_Materials.tar.gz › ESAT-esat_v0.1/doc/html/classumms_1_1core_1_1scripture_1_1_oriented_chromosome_transcript_graph-members.html]

ESAT: Member List


ESAT


- Main Page
- Related Pages
- Packages
- Classes
- Files

- Class List
- Class Index
- Class Hierarchy
- Class Members

All Classes Namespaces Files Functions Variables Enumerator Pages

- umms
- core
- scripture
- OrientedChromosomeTranscriptGraph

umms.core.scripture.OrientedChromosomeTranscriptGraph Member List

This is the complete list of members for umms.core.scripture.OrientedChromosomeTranscriptGraph, including all inherited members.

|  |  |  |
| --- | --- | --- |
| addAnnotationToGraph(Annotation a) | umms.core.scripture.OrientedChromosomeTranscriptGraph |  |
| addEdge(Annotation v1, Annotation v2) | umms.core.scripture.OrientedChromosomeTranscriptGraph |  |
| addEdge(Annotation v1, Annotation v2, boolean augmentCount) | umms.core.scripture.OrientedChromosomeTranscriptGraph |  |
| addVertex(Annotation v) | umms.core.scripture.OrientedChromosomeTranscriptGraph |  |
| connectVertexToGraph(Annotation v) | umms.core.scripture.OrientedChromosomeTranscriptGraph |  |
| getNodePair(TranscriptGraphEdge edge) | umms.core.scripture.OrientedChromosomeTranscriptGraph |  |
| getOrphanPaths() | umms.core.scripture.OrientedChromosomeTranscriptGraph |  |
| getOrphanVertices() | umms.core.scripture.OrientedChromosomeTranscriptGraph |  |
| getPaths() | umms.core.scripture.OrientedChromosomeTranscriptGraph |  |
| getSinkVertices() | umms.core.scripture.OrientedChromosomeTranscriptGraph |  |
| getSourceVertices() | umms.core.scripture.OrientedChromosomeTranscriptGraph |  |
| OrientedChromosomeTranscriptGraph(EdgeFactory< Annotation, TranscriptGraphEdge > edgeFactory) | umms.core.scripture.OrientedChromosomeTranscriptGraph | protected |
| OrientedChromosomeTranscriptGraph(String name, String orientation) | umms.core.scripture.OrientedChromosomeTranscriptGraph |  |
| pathToGene(GraphPath< Annotation, TranscriptGraphEdge > gp) | umms.core.scripture.OrientedChromosomeTranscriptGraph | static |
| writeGraph(String save, Alignments region, boolean setUpShortExonLabels, String graphName) | umms.core.scripture.OrientedChromosomeTranscriptGraph |  |


---

Generated on Fri Aug 15 2014 15:11:15 for ESAT by  

 1.8.7
